# Male circumcision uptake and misperceived norms about male circumcision: Cross-sectional, population-based study in rural Uganda

**DOI:** 10.7189/jogh.13.04149

**Published:** 2023-12-20

**Authors:** Jessica M Perkins, Bernard Kakuhikire, Charles Baguma, Sehee Jeon, Sarah F Walker, Rohit Dongre, Viola Kyokunda, Mercy Juliet, Emily N Satinsky, Alison B Comfort, Mark J Siedner, Scholastic Ashaba, Alexander C Tsai

**Affiliations:** 1 Department of Human and Organizational Development, Peabody College, Vanderbilt University, Nashville, Tennessee, USA; 2 Vanderbilt Institute for Global Health, Vanderbilt University Medical Center, Nashville, Tennessee, USA; 3 Mbarara University of Science and Technology, Mbarara, Uganda; 4 Department of Psychology, University of Southern California, Los Angeles, California, USA; 5 Center for Global Health, Massachusetts General Hospital, Boston, Massachusetts, USA; 6 Bixby Center for Global Reproductive Health, University of California, San Franciso, California, USA; 7 Harvard Medical School, Boston, Massachusetts, USA; 8 Mongan Institute, Massachusetts General Hospital, Boston, Massachusetts, USA

## Abstract

**Background:**

Over the past decade, 15 high-priority countries in eastern and southern Africa have promoted voluntary medical male circumcision for human immunodeficiency virus (HIV) and sexually transmitted infection (STI) prevention. The prevalence of male circumcision in Uganda nearly doubled from 26% in 2011 to 43% in 2016, but remains below the 2020 target level. Little is known about how common male circumcision is perceived to be, how accurate such perceptions are, and whether they are associated with men’s own circumcision uptake.

**Methods:**

We conducted a cross-sectional study of all adult residents of eight villages in Rwampara District, southwestern Uganda in 2020-2022. We elicited their perceptions of the adult male circumcision prevalence within their village: >50% (most men), 10% to <50% (some), <10%, (few to none), or do not know. We compared their perceived norms to the aggregated prevalence of circumcision reported in these villages. We used a modified multivariable Poisson regression model to estimate the association between perceived norms and personal circumcision uptake among men.

**Results:**

We surveyed 1566 participants (91% response rate): 698 men and 868 women. Among the men, 167 (27%) reported being circumcised, including 167/444 (38%) men <50 years of age. Approximately one-fourth of the population (189 (27%) men and 177 (20%) women) believed that few to no men in their own village had been circumcised. In a multivariable regression model, men who underestimated the prevalence of male circumcision were less likely to be circumcised themselves (adjusted relative risk (aRR) = 0.51; 95% confidence interval (CI) = 0.37-0.83).

**Conclusions:**

In this population-based study in rural Uganda, one-fourth of men underestimated the prevalence of male circumcision. Men who underestimated the extent of circumcision uptake were themselves less likely to be circumcised. If the observed association is causal and underestimates within the population contribute to low uptake, then interventions correcting these misperceived norms could increase uptake of voluntary medical male circumcision.

In 2007, the World Health Organization (WHO) and the Joint United Nations Programme on human immunodeficiency virus/acquired immune deficiency syndrome (HIV/AIDS) recommended increasing voluntary medical male circumcision (VMMC) uptake in populations at high risk of HIV infection, especially in HIV-endemic countries [1]. Studies had indicated that VMMC uptake was associated with reduced HIV acquisition risk among men [2-6] and, indirectly, among women [7,8]. Additionally, VMMC uptake was associated with a reduced risk of acquiring other sexually transmitted infections such as syphilis, herpes simplex virus type 2, penile human papillomavirus, and cervical cancer [9-13]. Consequently, governments and international organisations began supporting efforts to enhance VMMC uptake as a part of HIV prevention efforts [7,14], with recent research continually indicating its efficiency in this regard [15-18]. VMMC uptake reduces risk of infection by removing penile tissue that may be more susceptible to infection and by affecting the penile immune and microbial environment [19].

In 2016, the WHO set a target of achieving an additional 25 million VMMCs performed among boys and men across 15 “high-priority” countries in eastern and southern Africa by 2020 [20]. Although uptake increased [6], the cumulative number of VMMCs fell seven million short of the target for 2020, with uneven progress between countries and age groups [21]. For example, VMMC uptake is still much lower in Uganda than in other high priority countries [22,23] and is thus insufficient to translate into meaningful reductions in HIV incidence. Nationwide estimates indicate that the prevalence of circumcision among men aged 15-49 years had increased only to 43% by 2016-2017 [24,25], leaving a need for greater uptake among men at high risk of HIV acquisition and among men and boys aged ≥15 years [26]. Critically, increasing VMMC uptake requires novel implementation strategies, especially those that do not stigmatise uncircumcised individuals [27].

Several studies in southern Africa have found associations between men’s beliefs about what their social contacts would support and their personal intention to get circumcised. For example, young uncircumcised men in Eswatini were more likely to report a personal intention to get circumcised if they thought that their friends, parents, or partner encouraged male circumcision and if they thought that most of their male friends were circumcised [28]. Similarly, young uncircumcised men in Zimbabwe were more likely to report a personal intention to get circumcised if they believed that their mother would encourage male circumcision and if they believed that their male friends would get circumcised [29]. A recent systematic review concluded that familial and peer support for male circumcision facilitates uptake of VMMC [30]. Likewise, hearing about circumcision promotion from influential people such as religious leaders or a peer who underwent VMMC is also associated with circumcision uptake [31,32].

Theoretical and empirical research has shown that perceived descriptive norms (i.e. what individuals think most other people do) influence one’s own behaviors and beliefs [33-40]. However, individuals often misperceive local norms: they tend to underestimate the extent to which health-promoting behaviours are normative and overestimate the extent to which health-risk behaviours are normative [41-47]. Many factors contribute to this misalignment between perceived norms and actual local norms: lack of conversation with relevant peers about such behaviors or beliefs, lack of visible cues in the local environment about health-promoting behaviors, greater visibility of high risk behavior on social media, and biases in conversational, memory, and psychological inference processes [48,49]. Critically, health-promoting behaviors may be invisible due to associated stigma or local taboos preventing conversation, or because the behaviors are relatively private.

Studies have not yet assessed the accuracy of perceived norms about male circumcision nor the extent to which they can be harnessed to increase VMMC uptake in rural Uganda and in similar contexts. Recent studies on HIV prevention, substance use, violence, and other health-related behaviors in Uganda and South Africa have found that individuals often overestimate the prevalence of peer behaviors that increase the risk of HIV acquisition and transmission (e.g. avoiding testing, substance use, condomless sex, intimate partner violence, and non-adherence to antiretroviral medications) and that these misperceptions are associated with personal behavior [50-58].

We aimed to investigate perceptions about the local prevalence of male circumcision among all men and women across eight villages in rural Uganda. We compared participants’ beliefs about male circumcision prevalence (in their village) to the actual village-level prevalence of male circumcision. We then sought to estimate the association between these perceptions and men’s personal circumcision status. If many people misperceive local norms for male circumcision, then an opportunity would exist to assess whether correcting misperceptions would promote VMMC uptake. Many studies on other health behaviors have found that changing perceived norms leads to behavioral change [48,59-65].

## METHODS

### Study setting and design

We conducted a cross-sectional, whole-population study targeting all residents aged ≥18 years within eight villages in a rural, administrative parish in Rwampara District, southwestern Uganda, located approximately 20 km from Mbarara City. We selected this parish in collaboration with local leaders due to its tractable population, geographic size, and similarity to other rural areas in Uganda where most Ugandans reside [66]. Specifically, 75% of people in Uganda (and most people in other eastern and southern African countries) reside in local economies featuring agricultural and small-scale trading/enterprise, household food and water insecurity, and limited access to electricity and piped water [66-70]. The study population characteristics were also similar to national characteristics. For example, most adults in Uganda are married, have less than secondary education completed, and are between 18 and 30 years old [66,71,72]. Additionally, the setting resembles other areas in the 15 priority countries targeted for VMMC uptake in eastern and southern Africa.

### Study procedures

Research assistants who spoke the local language (Runyankore) gathered data in 2020-2022. Using a continuously updated parish census list of all age-eligible adult residents, they contacted all age-eligible residents of the targeted villages, if the residents were not incapacitated/intoxicated at the time of data collection. The research assistants asked for informed consent, obtaining participants’ signatures or thumbprints (for those unable to write). They then conducted one-on-one survey-based interviews and recorded survey responses using a computer-assisted tool. If procedures could not be conducted in person (typically in or near a participant’s home) due to coronavirus disease 2019 pandemic restrictions, the research assistants obtained consent and conducted data collection over the phone. All individuals who participated in the survey interview (in person or by phone) received their choice of a kilogram of sugar or a bar of soap (per local norms) for their time.

### Measures

The survey questions were written in English, translated into Runyankore, and then back-translated to English to verify the translation’s fidelity to the intended meaning. Question piloting and translation followed an iterative process. One question elicited from each man whether he was circumcised (yes/no), while another elicited from both men and women their estimates of the male circumcision prevalence in their village (i.e. their perceived norm about male circumcision uptake). Specifically, they were asked how many men in their own village were circumcised, using a 4-point Likert-type scale ranging from “all or almost all men (>90%)”, “more than half of men but fewer than 90%”, “fewer than half of men but more than 10%”, “very few or no men (<10%)”, or “do not know”. Pre-testing suggested that participants easily understood “Other adult men in your village” as the reference or comparison group for this question. Therefore, that group was set as the social reference group for identifying local norms [73,74]. Other studies conducted in this setting have used similar wording to capture perceptions about local norms. In the remainder of this manuscript, we use shorthand to refer to these perceived norm response categories as “most” (combining “all or almost all” and “more than half”), “some”, and “few”.

### Additional covariates

Male circumcision uptake varies by sociodemographic characteristics [22,75-82], HIV testing history [32,80,81,83], knowledge of one’s own HIV status [82], and condomless sexual activity [75]. We thus assessed several additional factors, including having had condomless sex with a non-spousal partner in the past year, having had a sexually transmitted infection (STI) in the past year, having been tested for HIV in the past year, perceived personal HIV risk (none, low, medium, or high), and HIV status. Sociodemographic variables included age, marital status (married/cohabiting vs divorced/separated/single), religion (Protestant, Catholic, Muslim, other), education (completed primary vs did not), and household wealth quintile. To measure household wealth, we created a household asset index by conducting a principal components analysis on 26 separate variables representing household assets and housing characteristics (no missing data). We retained the first principal component to define the wealth index and then split it into quintiles [84,85].

### Statistical analysis

To quantify the extent of misperceived norms, we compared respondents’ perceptions of the village-level prevalence of male circumcision to the actual village-level prevalence of self-reported circumcision among men. We calculated the prevalence of respondents who underestimated male circumcision prevalence and stratified it by circumcision status among men and sociodemographic subgroups.

We estimated the association between respondents’ perceptions of the village-level prevalence of male circumcision and self-reported circumcision among men <50 years of age and then re-estimated this association among all men. To do so, we fitted modified multivariable Poisson regression models specifying personal circumcision status as the dependent variable. With a binary dependent variable, the modified Poisson regression model has been shown to yield estimated incidence rate ratios that can be interpreted straightforwardly as relative risk ratios [86]. The models adjusted for HIV perceived risk and status, history of HIV testing, any STI in past year, condomless sex with nonspousal partner in past year, age, marital status, education, wealth, religion, and number of household members. We excluded Muslims in both models because all men who identified as Muslim reported being circumcised. We used cluster-correlated robust estimates of variance to account for clustering of observations by village [87], with *P* < 0.05 considered as statistically significant. We also calculated the predicted probabilities of being circumcised by perception categories. Finally, we used methods proposed by Vanderweele & Ding to calculate the e-value [88,89], a minimum strength of association (on the risk ratio scale) that an unobserved confounder would need to have with both the exposure (perception) and the outcome (circumcision) to completely account for the estimated association, conditional upon the included covariates. A large e-value suggests that potential confounding would need to be very strong in order to sufficiently explain away the observed association. We conducted all analyses with Stata, version 16 (StataCorp LLC, College Station, Texas, USA).

## RESULTS

Among 1723 people who were eligible for study participation, 1566 were interviewed (90.9% response rate), of whom 698 (45%) were men. The mean age across the full population was 42 years (standard deviation (SD) = 16). Most participants (940 (60%)) had completed primary education or more, and most were married/cohabiting as if married (1024 (65%)). Twenty-two participants (1%) identified as Muslim. Overall, 767 (49%) had been tested for HIV in the past 12 months, 180 (11%) reported an HIV-positive status, 75 (5%) had an STI in the past 12 months, 179 (11%) had condomless sex with a non-spousal partner in the past 12 months, and 220 (14%) perceived their personal HIV risk to be medium/high.

Thirty-eight men did not report their circumcision status: 17 refused to answer and 21 had never had sex and were accidentally not asked about their circumcision status due to a logic branching error during data collection. Among the 660 men with a reported circumcision status, 191 (27%) reported being circumcised. This prevalence ranged from 23% to 37% across the eight villages.

Among 444 male participants who were <50 years of age and who provided a response about their circumcision status, 167 (38%) reported that they were circumcised. Village-level circumcision rates ranged from 27% to 51%. The male circumcision prevalence was higher among younger age groups. For example, almost half of men aged 18-25 years old were circumcised (n = 40 (48%)). The prevalence was lower among men who had not completed primary school (**Table 1**).

**Table 1:** Characteristics of all male residents, and those who reported being circumcised, aged 18-50-years-old across eight villages in Rwampara District, southwest Uganda*

	Men in study <50 years old†	Men who reported being circumcised
**Total**	444 (100)	167 (38)
**Age in years**		
18-25	84 (19)	40 (48)
26-35	160 (36)	64 (40)
36-45	152 (34)	49 (32)
46-55	48 (11)	14 (29)
**Marital status**		
Not married	147 (33)	57 (39)
Married/cohabiting as if married	297 (67)	110 (37)
**Religion**		
Catholic	93 (21)	40 (43)
Muslim	7 (2)	7 (100)
Protestant	321 (72)	110 (34)
Other (not religious, seventh day adventist)	23 (5)	10 (43)
**Education**		
Less than primary education	112 (25)	27 (24)
Completed primary education	332 (75)	140 (42)
**Household asset wealth**		
1st (poorest)	83 (19)	28 (34)
2nd	99 (22)	36 (36)
3rd	90 (20)	34 (38)
4th	87 (20)	31 (36)
5th (least poor)	85 (19)	38 (45)
**Was tested for HIV in past 12 mo**		
No	227 (51)	74 (33)
Yes	217 (49)	93 (43)
**Had an STI in past 12 mo**		
No	411 (93)	153 (37)
Yes	33 (7)	14 (42)
**Had condomless sex with a non-spouse partner in past 12 mo**		
No	371 (84)	136 (37)
Yes	73 (16)	31 (42)
**Perceived personal HIV risk**		
Identifid as HIV-positive	32 (7)	9 (28)
Identified as HIV-negative/unknown status with perceived no/low HIV risk	353 (80)	137 (39)
Identified HIV-negative/unknown status with perceived medium/high HIV risk	55 (13)	20 (36)

*Values presented as n (%) unless specified otherwise.

†Circumcision status for 28 participants was unknown and thus is not included in this table: nine refused to answer and 19 were accidentally not asked the question after indicating that they had never had sex.

### Extent of underestimated norms about male circumcision

Among the 469 men who had not been circumcised, 154 (33%) thought that few to no men had been circumcised and 47 (10%) did not know. Among the 191 men who had been circumcised, 26 (14%) thought that few to no men had been circumcised and nine (5%) did not know. Among 868 women, 177 (20%) thought that few to no men had been circumcised and 220 (25%) did not know. Overall, 366 of 1566 participants (23%) incorrectly thought that few men in their villages had been circumcised, while 287 (18%) reported not knowing this information. Misperceiving that few or no village men had been circumcised varied in prevalence from 10% to 35% across sex-specific sociodemographic and HIV risk categories (**Table 2**). The combined prevalence of underestimation and “do not know” responses varied from 36% to 47% across villages.

**Table 2:** Misperceived norms about male circumcision in own village among adults across eight villages in Rwampara District, southwest Uganda (n = 1566)*

	Number of male study participants	Men who inccorectly thought few men are circumcised	Men who did not know how many men are circumcised	Number of female study participants	Women who incorrectly thought few men are circumcised	Women who did not know how many men are circumcised
**Total**	698	189 (27)	67 (10)	868	177 (20)	220 (26)
**Age in years**						
17-25	102	20 (20)	7 (7)	139	37 (27)	30 (22)
26-35	164	39 (24)	8 (5)	213	48 (23)	36 (17)
36-45	157	55 (35)	10 (6)	182	41 (23)	38 (21)
46-55	142	37 (26)	15 (11)	146	23 (16)	29 (20)
≥56	130	38 (29)	26 (20)	174	28 (16)	78 (46)
**Marital status**						
Not married and not cohabiting	205	50 (24)	22 (11)	337	71 (21)	109 (33)
Married/cohabiting as if married	493	139 (28)	45 (9)	531	106 (20)	111 (21)
**Religion**						
Catholic	155	40 (26)	17 (11)	189	39 (21)	44 (23)
Muslim	9	2 (22)	0 (0)	13	2 (15)	4 (31)
Protestant	505	140 (28)	48 (10)	617	123 (20)	157 (26)
Other (Not religious; Seventh-Day Adventist; Born-again Pentecostal)	29	7 (24)	2 (7)	49	13 (27)	15 (31)
**Education**						
None/some primary education	224	65 (29)	29 (13)	402	63 (16)	126 (32)
Completed primary education or more	474	124 (26)	38 (8)	466	114 (24)	94 (20)
**Household asset wealth**						
1st quintile (poorest)	111	33 (30)	6 (5)	202	40 (20)	62 (31)
2nd quintile	138	41 (30)	10 (7)	175	36 (21)	35 (20)
3rd quintile	142	31 (22)	15 (11)	172	32 (19)	43 (25)
4th quintile	152	39 (26)	20 (13)	161	37 (23)	40 (25)
5th quintile (least poor)	155	45 (29)	16 (10)	158	32 (20)	40 (25)
**Had been tested for HIV in past 12 months**						
No	383	106 (28)	44 (12)	416	65 (16)	130 (32)
Yes	315	83 (26)	23 (7)	452	112 (25)	90 (20)
**Had an STI in past 12 momths**						
No	637	176 (28)	65 (10)	803	157 (20)	202 (25)
Yes	40	8 (20)	0 (0)	35	8 (23)	12 (34)
**Had condomless sex with a non-spouse partner in past 12 months**						
No	580	166 (29)	56 (10)	756	148 (20%)	187 (25)
Yes	97	18 (19)	9 (9)	82	17 (21)	27 (33)
**Perceived personal HIV risk**						
Identifid as HIV-positive	67	22 (33)	6 (9)	113	24 (21)	21 (19)
Identified as HIV-negative/unknown status with perceived no/low HIV risk	546	138 (25)	51 (9)	598	126 (21)	164 (28)
Identified HIV-negative/unknown status with perceived medium/high HIV risk	77	26 (34)	8 (11)	143	26 (18)	33 (23)

HIV – human immunodeficiency virus, STI – sexually transmitted infection

*Values presented as n (%) unless specified otherwise.

### Perceived norms as correlates of personal circumcision status

Among men <50 years of age and who did not identify as Muslim (n = 433), those who perceived that most men in their villages had been circumcised were more likely to be circumcised than men who perceived that some men had been circumcised (adjusted relative risk (aRR) = 1.67; 95% CI = 1.20-2.30, *P* = 0.002). The associated e-value was 2.73.

**Figure 1:**
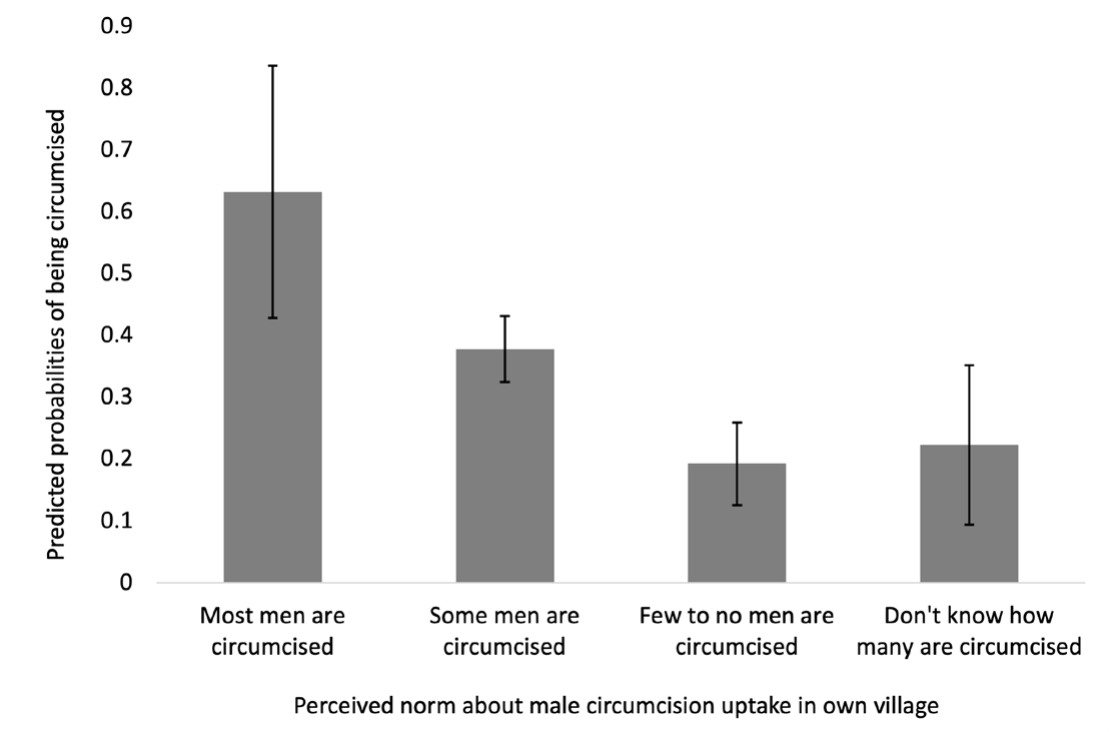
Predicted probabilities of being circumcised among all resident men <50 years of age across eight villages in Rwampara District, southwestern Uganda, stratified by their perception of the male circumcision prevalence in their own village after adjusting for other factors. Error bars represent the 95% confidence interval for the predicted probability.

Men who perceived that few to no men in their villages had been circumcised were less likely to be circumcised (aRR = 0.51; 95% CI, 0.35-0.74, *P* < 0.001). Similarly, men who reported that they did not know how many other men in their village were circumcised were also less likely to be circumcised, although the estimate was imprecise (aRR = 0.59; 95% CI = 0.33-1.06, *P* = 0.076). We present the predicted probabilities of being circumcised (and their 95% confidence intervals) by perception categories in **Figure 1**.

While the risk of having been circumcised appeared to decrease with age, estimates between age and circumcision status were not precise. Similarly, while men who had finished primary education or more had a greater risk of having been circumcised, the estimate was imprecise (aRR = 1.54; 95% CI = 0.98-2.42, *P* = 0.064). None of the HIV risk factors nor any of the other sociodemographic factors were associated with participant circumcision status (**Table 3**). Results from the sensitivity analysis including all men indicate that the pattern of associations were similar to those found in main analyses (Table S1 in the **Online Supplementary Document**).

**Table 3:** Modified multivariable Poisson regression model estimating associations between the perceived norm about circumcision status among men in one’s village and being personally circumcised among almost all resident adult men <50 y old (excluding Muslim men) across eight villages in Rwampara District, southwestern Uganda (n = 433 men)

Yes, circumcised		
	**aRR (95% CI)**	***P*-value**
**Perceived norm about male circumcision uptake in own village**		
Most men are circumcised (i.e. >50%)	1.67 (1.20-2.33)	0.002
Some men are circumcised (i.e. 10% to <50%)	ref	-
Few men are circumcised (i.e. 0 to <10%)	0.51 (0.35-0.75)	0.001
Don't know how many men are circumcised	0.59 (0.33-1.06)	0.076
**Age (years)**		
18-25	ref	-
26-35	0.91 (0.68-1.22)	0.520
36-45	0.81 (0.63-1.03)	0.086
46-55	0.72 (0.44-1.17)	0.182
**Married/cohabiting (vs other)**	1.17 (0.93-1.48)	0.180
**Religion**		
Catholic	ref	
Protestant	0.86 (0.62-1.19)	0.375
Other	1.10 (0.63-1.93)	0.727
**Completed primary education or more (vs did not)**	1.54 (0.98-2.42)	0.062
**Household asset quintile**		
1st quintile (poorest)	ref	-
2nd quintile	0.96 (0.72-1.27)	0.758
3rd quintile	0.88 (0.52-1.49)	0.626
4th quintile	0.86 (0.57-1.28)	0.451
5th quintile (least poor)	1.17 (0.75-1.82)	0.480
**Tested for HIV in past 12 mo (vs did not)**	1.18 (0.80-1.73)	0.408
**Had an STI in past 12 mo (vs did not)**	1.10 (0.61-1.98)	0.759
**Had condomless sex with a non-spouse partner in past 12 mo (vs did not)**	1.02 (0.84-1.23)	0.872
**Perceived personal HIV risk**		
Identifid as HIV-positive	0.88 (0.58-1.34)	0.555
Identified as HIV-negative/unknown status with perceived no/low HIV risk	ref	-
Identified HIV-negative/unknown status with perceived medium/high HIV risk	1.07 (0.87-1.32)	0.520

aRR – adjusted relative risk, CI – confidence interval, ref – reference group, HIV – human immunodeficiency virus, STI – sexually transmitted infection

## DISCUSSION

In this population-based study across eight villages in rural Uganda, we found that many men and women underestimated the local prevalence of circumcision among men. They incorrectly thought that circumcision was extremely rare among men, even though 27% of men reported being circumcised (as did 38% of men aged 18-49). These misperceptions were present across all population subgroups including by circumcision status among men.

The public health importance of this finding is that men who misperceived norms about circumcision (i.e. underestimated the prevalence of circumcision among men in their villages) were less likely to be circumcised themselves. Importantly, there may have been other potential confounders not included in the multivariable regression models. For example, having poor HIV prevention knowledge could be correlated with low VMMC uptake and with greater misperceptions about the extent to which circumcision is normative. A regression model estimating the association between misperceptions and VMMC uptake that did not adjust for HIV prevention knowledge could therefore yield estimates of the association that are biased away from the null. Based on the e-value analysis, such an unobserved confounder would need to have an association with both circumcision uptake and perceiving that most men were circumcised of 2.73 on the risk ratio scale, conditional on the measured covariates, to explain away the association observed in our study.

Based on other studies in the region, we expect this possibility to be unlikely. For example, in a population-based study of men in eastern and southern Africa, having poor HIV prevention knowledge was associated with reduced odds of circumcision, but the magnitude of this association (adjusted odds ratio (aOR) = 0.83 overall, aOR = 0.53 in the Rwanda subsample, aOR = 1.26 in the Kenya subsample) would not have been large enough to explain away the estimates we obtained [90].

Our findings are in line with previously published studies showing that misperceived norms are an important driver of outcomes related to HIV prevention and treatment, such as HIV testing, adherence to antiretroviral therapy, condom use, and HIV-related stigma, both in rural Uganda and elsewhere in eastern and southern Africa [50,51,54,58,91]. Taken together, these findings indicates that a social norms approach [49,92,93] to VMMC uptake would be applicable in contexts where a substantial, but not sufficient (i.e. from a public health perspective) part of the male population is circumcised, and where male circumcision has increased over time.

For example, uncircumcised men and their partners who incorrectly believe male circumcision to be rare could receive personalised normative feedback [94-97] about actual circumcision rates in the population. Additionally, a social norms campaign [98-100] targeting the whole population could emphasize VMMC as a trending norm [101] in the local context and in Uganda more broadly. These strategies would aim not to address health education or behavior change directly, but simply to correct underestimates and reinforce support for male circumcision uptake. Messages based on factual information might include: “In 2021, more than one out of every three men in this village chose to get circumcised”, or “The number of men choosing to get circumcised continues to grow. Now almost half of men aged 18 to 35 years in your village have been circumcised”. Health care providers and community health workers could share this information during routine visits or as part of other HIV- or contraception-related interventions. Similarly, local leaders could receive training on trending norms information and facilitate discussions or one-on-one conversations [31]. Local norms could be visually displayed at the entrances of clinics offering VMMC services [102] or publicised through radio or edu-entertainment messages [103,104]. Studies on other topics have found that enhancing the salience and visibility of local health-promoting norms can shift individuals’ expectations about typical and acceptable behavior and attitudes when misalignment between perceptions and actual norms exists, and in turn, prompt behavior change [48,59-65,91,100,105]. Additionally, increasing awareness of trending norms (behaviors increasing in prevalence) can also prompt behavior change [101,106,107].

Addressing incorrect beliefs about circumcision being rare among uncircumcised men (i.e. correcting their state of false consensus) [108] may encourage these men to conform with prevailing norms and to seek ways to overcome existing barriers to circumcision. Increased awareness of the actual trending norm may also weaken negative beliefs about circumcision and encourage action on previous, unfulfilled intentions. Furthermore, rectifying prevalence underestimates among circumcised men (i.e. correcting their state of pluralistic ignorance) [109] may prompt them to share their experiences with others, as they realize that their behavior aligns with the trend. They might be inclined to publicly advocate for VMMC or support others in seeking options to do so. More information regarding the higher-than-expected and increasing circumcision prevalence could help mitigate stigmatising beliefs and misconceptions, especially if paired with health education about the benefits of male circumcision.

Correcting underestimates and ignorance among women about male circumcision uptake and its upward trend may also indirectly lead to VMMC uptake. Mothers, sisters, and female partners often influence men’s circumcision decisions [28,29,110-113]. Addressing their underestimates may reduce stigma toward male circumcision, increase conversation in support of circumcision, increase support for male partners to navigate any access barriers, and prevent spread of norm misperceptions. Pressure from female partners to undergo circumcision resulted in men in Uganda undergoing VMMC [113]. Men reported that their partners believed in the importance of circumcision after hearing about it from radios, newspapers, and health services [113].

This study has limitations in addition to the possible confounding by unmeasured covariates mentioned above. First, our findings may not be generalisable beyond the parish studied. However, the participants surveyed represent more than 90% of the adult parish population, and the study context is similar to rural areas across Uganda and in eastern and southern Africa. Second, our primary outcome (circumcision status) was based on self-reporting. Based on other studies conducted throughout eastern Africa, we expect self-reported circumcision status to correlate highly with circumcision status ascertained through physical examination [114]. Moreover, the prevalence of circumcision among 18-49-year-old men in our sample (38%) was slightly larger than the prevalence of circumcision among 15-49-year-old men in the 2016 Uganda Demographic and Health Survey (i.e. 26%) [66], which is consistent with the increasing uptake of circumcision at the population level within these groups. Despite these limitations, this study provides key evidence for future research on the extent to which highlighting VMMC uptake as an increasingly common decision can motivate uptake.

## CONCLUSION

VMMC uptake continues to be an important cost-effective HIV prevention strategy. In this population-based study in rural Uganda, one-third to almost half of adults in every village either underestimated or did not know how many men in their own village had been circumcised. Misperceptions were present across all population subgroups, and men who had these misperceptions were less likely to report being circumcised themselves. These findings collectively indicate the potential for an opportunity to motivate VMMC uptake by addressing any underestimates of male circumcision uptake in an HIV-endemic setting where numerous men in the general community are at high-risk for HIV acquisition. Future research should assess the extent to which changing underestimated norms among uncircumcised men directly motivates VMMC uptake. Additionally, changing underestimated norms among women and circumcised men may increase conversation and support for others to get circumcised. Male circumcision in Uganda has trended upward since 2011. Providing information about the rising trend in male circumcision rates in this context and correcting prevalence underestimates will enhance VMMC uptake visibility and salience. This type of social norms approach strategy targeting both men and women to promote VMMC uptake could complement efforts to educate individuals about circumcision and its health benefits.

## Additional material


Online Supplementary Document
