# Supplementary material for: Male circumcision uptake and misperceived norms about male circumcision: Cross-sectional, population-based study in rural Uganda
Source: J Glob Health. 2023 Dec 20;13:04149. doi: 10.7189/jogh.13.04149 (PMC10731132; doi:10.7189/jogh.13.04149)
Supplement: Online Supplementary Document [file jogh-13-04149-s001.pdf]

Table S1. Modified multivariable Poisson regression model estimating associations between the perceived norm about circumcision status among men in one's village and being personally circumcised among almost all resident adult men across eight villages in Rwampara District, southwestern Uganda (n=649 men).

	Yes, circumcised		
	aRR	(95% CI)	p-value
Perceived norm about male circumcision uptake in own village			
Most men are circumcised (i.e., >50%)	1.81	(1.41 – 2.32)	<0.001
Some men are circumcised (i.e., 10% to <50%)	REF	-	-
Few men are circumcised (i.e., 0 to <10%)	0.53	(0.35 – 0.79)	0.002
Don't know how many men are circumcised	0.73	(0.45 – 1.19)	0.209
Age (years)			
18-25	REF	-	-
26-35	0.95	(0.74 – 1.23)	0.699
36-45	0.84	(0.67 – 1.04)	0.105
46-55	0.52	(0.40 – 0.68)	<0.001
56+	0.23	(0.15 – 0.36)	<0.001
Married / cohabiting (vs. other)	1.09	(0.89 – 1.34)	0.419
Religion			
Catholic	REF	-	-
Muslim	2.91	(2.12 – 4.00)	<0.001
Protestant	0.88	(0.62 – 1.24)	0.453
Other	1.33	(0.84 – 2.10)	0.229
Completed primary education or more (vs. did not)	1.65	(1.13 – 2.40)	0.009
Household asset quintile			
1st quintile (poorest)	REF	-	-
2nd quintile	0.95	(0.69 – 1.30)	0.737
3rd quintile	0.87	(0.53 – 1.43)	0.589
4th quintile	0.94	(0.67 – 1.30)	0.693
5th quintile (least poor)	1.23	(0.83 – 1.83)	0.297
Tested for HIV in past 12 months (vs. did not)	1.09	(0.82 – 1.45)	0.538
Had an STI in past 12 months (vs. did not)	1.01	(0.55 – 1.84)	0.986
Had condomless sex with a non-spouse partner in past 12 months (vs. did not)	1.08	(0.91 – 1.29)	0.356
Perceived personal HIV risk			
Identifid as HIV-positive	1.14	(0.75 – 1.73)	0.539
Identified as HIV-negative/unknown status with perceived no/low HIV risk	REF	-	-
Identified HIV-negative/unknown status with perceived medium/high HIV risk	1.03	(0.92 – 1.16)	0.598

aRR = adjusted relative risk ratio, CI = confidence interval, REF = reference group
